# Structure and Dynamics of Highly Attractive Polymer Nanocomposites in the Semi-Dilute Regime: The Role of Interfacial Domains and Bridging Chains

**DOI:** 10.3390/polym13162749

**Published:** 2021-08-16

**Authors:** Emmanuel N. Skountzos, Katerina S. Karadima, Vlasis G. Mavrantzas

**Affiliations:** 1 Department of Chemical Engineering, University of Patras and FORTH-ICE/HT, GR 26504 Patras, Greece; skountzos@chemeng.upatras.gr (E.N.S.); kkaradima@chemeng.upatras.gr (K.S.K.); 2 Particle Technology Laboratory, Department of Mechanical and Process Engineering, ETH Zürich, CH 8092 Zürich, Switzerland

**Keywords:** polymer nanocomposites, polymer network, molecular simulations, molecular dynamics, silica, PEG

## Abstract

Detailed molecular dynamics (MD) simulations are employed to study how the presence of adsorbed domains and nanoparticle bridging chains affect the structural, conformational, thermodynamic, and dynamic properties of attractive polymer nanocomposite melts in the semi-dilute regime. As a model system we have chosen an unentangled poly(ethylene glycol) (PEG) matrix containing amorphous spherical silica nanoparticles with different diameters and at different concentrations. Emphasis is placed on properties such as the polymer mass density profile around nanoparticles, the compressibility of the system, the mean squared end-to-end distance of PEG chains, their orientational and diffusive dynamics, the single chain form factor, and the scattering functions. Our analysis reveals a significant impact of the adsorbed, interfacial polymer on the microscopic dynamic and conformational properties of the nanocomposite, especially under conditions favoring higher surface-to-volume ratios (e.g., for small nanoparticle sizes at fixed nanoparticle loading, or for higher silica concentrations). Simultaneously, adsorbed polymer chains adopt graft-like conformations, a feature that allows them to considerably extend away from the nanoparticle surface to form bridges with other nanoparticles. These bridges drive the formation of a nanoparticle network whose strength (number of tie chains per nanoparticle) increases substantially with increasing concentration of the polymer matrix in nanoparticles, or with decreasing nanoparticle size at fixed nanoparticle concentration. The presence of hydroxyl groups at the ends of PEG chains plays a key role in the formation of the network. If hydroxyl groups are substituted by methoxy ones, the simulations reveal that the number of bridging chains per nanoparticle decreases dramatically, thus the network formed is less dense and less strong mechanically, and has a smaller impact on the properties of the nanocomposite. Our simulations predict further that the isothermal compressibility and thermal expansion coefficient of PEG-silica nanocomposites are significantly lower than those of pure PEG, with their values decreasing practically linear with increasing concentration of the nanocomposite in nanoparticles.

## 1. Introduction

Due to their high surface-to-volume ratios, nanoparticles when embedded in a polymer matrix can induce substantial changes to the physicochemical (dynamical, mechanical, barrier, optical, electrical etc.) properties of the material. Understanding the key mechanisms driving these changes can substantially guide the molecular design of new polymer nanocomposites with tailored properties for specific applications, as also summarized in several excellent reviews [1,2,3,4,5,6,7,8,9,10,11,12]. From a design point of view, important factors to consider when analyzing the properties of such systems are the size and concentration of the nanoparticles in the matrix and the nature of interactions (attractive, repulsive, or athermal) between nanoparticles and host polymer segments. According to Liu et al. [13], in the dilute regime, changes in the physicochemical properties of the polymer matrix are mainly due to interfacial effects from chain segments that are adsorbed tightly on the surface of the additives. In this regime, interfacial domains around each nanoparticle are distinct and relatively isolated, and do not overlap. In the high concentration regime, on the other hand, these interfacial domains overlap substantially, which can lead further to chain confinement effects driving strong alterations in the conformation and size in the entire population of matrix chains. Between these two limits, there is the semi-dilute regime where adsorbed polymer layers do not overlap but nanoparticles are already close enough for the matrix chains to be able to form contacts with more than one nanoparticle, thus creating bridges (connections) between them. It is understandable then that the conformational, and more importantly, the dynamic properties of the matrix will depend strongly on the degree of such nanoparticle bridging by tie polymer chains, as well as on the structure and conformation (density, packing, spatial extent) of the adsorbed layer around each nanoparticle. It is exactly on this regime where the present study focuses.

For relatively short polymer chains (e.g., with lengths below the characteristic entanglement molecular length), it is also understandable that functional groups present at the ends of polymer chains can have a strong effect on the mechanism of chain adsorption on the nanoparticle, which in turn depends crucially on the size of nanoparticles and their concentration in the polymer matrix, factors that also affect the capability of chains to link different nanoparticles [14].

As already alluded to above, a parameter that is quite useful in prioritizing interactions in polymer nanocomposites is the inter-particle distance *d*_inter_, defined as the minimum surface-to-surface separation between neighboring nanoparticles. Clearly, when *d*_inter_ becomes comparable to the size of polymer chains, interesting phenomena should be expected due to the interplay between adsorbed interfacial layers, alterations in polymer conformation, and development of an underlying microscopic structure (topological network) by bridging chains that link different nanoparticles. Baeza et al. [15] examined the collective dynamics of poly(2-vinyl pyridine) (P2VP) melts filled with spherical silica nanoparticles and observed the transition to a gel-like dynamics for nanoparticle loadings above a critical value where *d*_inter_ becomes comparable to the size of polymer chains; this favors the formation of a bridge-like network in the nanocomposite, with nanoparticles acting as nodes and P2VP chains as strands connecting different nanoparticles. The authors also reported that at extreme loadings of the matrix in silica nanoparticles, the temperature dependence of microscopic dynamics changes from a Williams-Landel-Ferry (WLF) to an Arhenius-like one. This was attributed to the fact that at such large volume fractions, the polymer bound layers that develop around nanoparticles start to percolate, thus dominating the network-like character of the nanocomposite at medium or lower silica concentrations.

For the same system, Chen et al. [16] have reported that the transition to gel-like dynamics depends strongly on the size of nanoparticles (thus, it can have an effect also in systems containing nanoparticles in lower concentrations but of smaller size). The authors proposed a model capable of describing data collected from rheological experiments with emphasis on the transition from a gel-like network to a glassy structure when the particles come close enough for their bound layers to overlap (the transition occurs when *d*_inter_ becomes smaller than the polymer Kuhn length).

Formation of a topological network driven by chains that bridge different nanoparticles and transition to a gel-like state has also been reported by Kim et al. [17] for poly(ethylene glycol) (PEG) matrices filled with spherical, amorphous silica nanoparticles; the authors studied also how such a transition depends on the molecular weight (MW) of the host chains. For the same system (PEG-silica), Anderson and Zukoski [18,19] reported a significant increase of the viscosity at high silica loadings, both for unentangled [18] and entangled [19] matrix chains.

In addition to direct experimental measurements, molecular simulations can also help address the nanoscale properties of polymer nanocomposites. Barbier et al. [20] reported a detailed study of low-MW PEG melts filled with small amorphous silica nanoparticles with the help of molecular dynamics (MD) simulations. Even though the overall size of polymer chains in the simulated nanocomposites remained unperturbed, microscopic dynamics were considerably slowed down compared to the bulk, due to the strong interaction of polymer chains with the filler [20]. The results were further found to depend on the chemistry of the terminal units (hydroxyl- versus methoxy-) of the host polymer chains. A very recent study by Skountzos et al. [21] revealed completely different conformational, structural, and dynamic behaviors between hydroxyl- and methoxy-terminated PEG matrices filled with amorphous silica nanoparticles. Hydroxyl-terminated PEG chains were found to adopt graft-like conformations by adsorbing on the silica nanoparticles by their end groups, whereas methoxy-terminated PEG chains were found to adsorb by lying on the nanoparticle along their entire backbone [21]. Additional simulation results [21] for the single chain intermediate coherent dynamic structure factor were in remarkable agreement with previously reported experimental data by Glomann et al. [22,23]. In an earlier study, Rissanou et al. [24] have reported that the size of PEG chains in their nanocomposites with amorphous silica nanoparticles remains unaltered compared to the pure melt; however, with increasing silica content, a non-negligible increase in the relative population of gauche conformational states for the COCC and OCCO backbone dihedral angles of the polymer was observed in the simulations. The authors further reported that no significant change in the conformation of the polymer matrix chains was observed upon switching from CH_3_- to OH- terminal units.

In addition to PEG-silica, several other polymer nanocomposite systems have been addressed with molecular simulations [20,21,24,25,26,27,28,29,30,31,32,33,34,35,36,37,38,39,40,41,42,43,44,45,46,47]. We mention, for example, the work of Ndoro et al. [40,41] on curvature effects in melts of poly(styrene) (PS) oligomers filled with spherical silica particles. The local PS density around the silica surface was found to decrease with increasing curvature [40,41] (i.e., for smaller nanoparticles); in addition, polymer chains were found to swell and their dynamics significantly slowed-down compared to the pure melt. These findings agree with the results of Mathioudakis et al. [39] from a new multiscale simulation approach that helped examine high-MW PS-silica nanocomposites. Similar conclusions have been reported by Pandey and Doxastakis [33] for a poly(ethylene) (PE) melt filled with very small (highly curved) silica nanoparticles. However, when silica particles were replaced by fullerene (C_60_) molecules of size considerably smaller than that of silica, the polymer density in the vicinity of C_60_ increased significantly.

In a similar conception, Karatrantos et al. [43] performed coarse-grained (CG) MD simulations in order to examine polymer conformations in polymer nanocomposites characterized by either strong or weak interactions of the spherical inclusions with the host matrix. For repulsive interactions, the size of the polymer chains remained completely unperturbed. In contrast, when attractive interactions were assumed, swollen polymer conformations were observed. The same authors investigated how the *R*_p_/*R*_g_ ratio (*R*_p_ stands for the nanoparticle radius and *R*_g_ for the polymer radius-of-gyration) affects the conformational properties and found that, when *R*_p_/*R*_g_ < 1, polymer chains significantly expand [43], especially at higher nanoparticle volume fractions.

Eslami et al. [31] employed detailed MD simulations to study poly(methyl methacrylate) (PMMA) oligomers filled with spherical silica nanoparticles (another highly attractive system). More recently, Behbahani et al. [25] have performed atomistic MD simulations to study poly(butadiene) (PB)-silica nanocomposites. Similar to previous works, they found that polymer chains close to silica nanoparticles tend to assume extended conformations by wrapping around the nanoparticles, but the phenomenon becomes less pronounced in higher-MW PB melts. The authors also reported that decreasing *d*_inter_ (e.g., by increasing the silica volume fraction) favors the formation of a bridge-like network, since more chains can adsorb on the nanoparticles or more chains can create bridges between nanoparticles.

Our goal in this work is to use MD simulations to provide additional significant insight into the structural and dynamic properties of highly attractive polymer nanocomposites with emphasis in the semi-dilute regime and the role of interfacial and bridging chains. Our work is a continuation of the work presented in ref. [21] on the conformation and dynamics of PEG-silica nanocomposites which was carried out at fixed filler volume fraction and fixed filler size, and demonstrated the important role of end groups on the microscopic dynamics of these nanocomposites. In the present work, we carry out a parametric analysis of the dependence of structural and dynamic properties of PEG-silica nanocomposites on silica nanoparticle volume fraction and size. We also present a detailed analysis of the topological features of the polymer network formed between PEG chains and silica nanoparticles, and how this network is affected by the presence of different groups (hydroxyl- versus methoxy-) at the ends of PEG chains. We also calculate two thermodynamic quantities, the isothermal compressibility and the thermal expansion coefficient, that are important to consider in actual applications of these nanocomposites in practice.

The rest of the paper is organized as follows. In Section 2, we discuss the systems simulated in this work and provide details concerning technicalities of the MD simulations. In Section 3, we present and discuss results referring to the local mass density profile and chain packing in the neighborhood of nanoparticles, the conformation of adsorbed and free PEG chains, the static structure factor, the single chain form factor and the self-intermediate scattering function of PEG chains, features of the nanoparticle network formed by chains bridging different nanoparticles, the orientational and translational dynamics of adsorbed and free PEG chains, and the compressibility of the nanocomposites. The paper concludes with Section 4 summarizing the most important findings of the work and discussing possible future research efforts.

## 2. Systems Studied and Simulation Details

**Figure 1:**
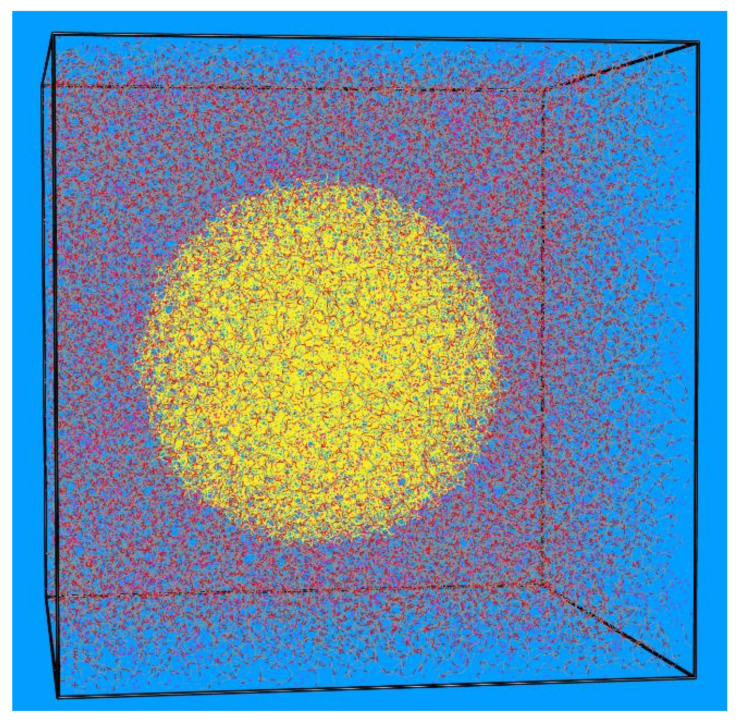
Typical atomistic configuration in box-relative coordinates of the d9_v15_PEG system (system 4 in Table 1) containing 750 PEG chains with degree of polymerization *N* = 41 and one spherical silica nanoparticle of diameter *d* = 9 nm in a cubic simulation cell with dimensions equal to 138 Å × 138 Å × 138 Å. Carbon, oxygen, hydrogen and silica atoms are shown with gray, red, white, and yellow color, respectively.

The polymer matrix consists of unentangled poly(ethylene glycol) chains with degree of polymerization (number of monomers per chain) *N* = 41 (implying a molecular weight equal to 1824.20 g mol^−1^) terminated with hydroxyl groups (i.e., PEG chains). Eight different model systems were examined, denoted as systems 1−8, with system 1 corresponding to the pure PEG melt and systems 2−8 to the PEG-silica nanocomposites. The molecular characteristics of these systems (number of PEG chains and total number of interacting units, nanoparticle diameter, nanoparticle volume fraction, and surface concentration of nanoparticles in silanol groups) and abbreviations concerning their notation can be found in Table 1. For example, with the name d5_v15_PEG we mean the nanocomposite that consists of PEG chains (i.e., chains terminated with hydroxyl groups) and contains silica nanoparticles with diameter *d* = 5 nm at volume fraction *v* = 15 *v*/*v*%. Detailed information concerning the construction of the amorphous silica nanoparticles and their surface concentration in silanol moieties (SiOH, Si(OH)_2_ and Si(OH)_3_ groups) can be found in ref. [21]. Initial structures were built by placing one silica particle in the simulation box and filling the remaining volume with PEG chains at the desired volume fraction *v* (in the range from 15 to 35 *v/v*%). By construction, our polymer nanocomposites are characterized by an ideal dispersion of nanoparticles in the polymer matrix, thus phenomena related with non-uniformities in nanoparticle dispersion or with nanoparticle agglomeration are absent. To examine the effect of the nature of the terminal groups on the structure of the network formed, we also simulated a system that is identical to the d5_v15_PEG one in Table 1, except that the hydroxyl terminal groups in the host polymer chains have been replaced by methoxy ones (system d5_v15_PEO in Table 1). A typical atomistic configuration from the atomistic simulations with system 4 (system d9_v15_PEG in Table 1) is shown in Figure 1.

Initial configurations were built with the Amorphous Builder module based on the work of ref. [48] integrated in the Scienomics MAPS software (Paris, France) [49], followed by static structure optimization to minimize the potential energy and obtain an equilibrium structure that is devoid of atom overlaps. The resulting minimum-potential energy configuration for each system was submitted to a long MD simulation in the isothermal-isobaric *npT* (constant number of atoms, *n*, pressure, *p*, and temperature, *T*) statistical ensemble at *T* = 413 K and *p* = 1 atm, conditions where PEG and its silica-based nanocomposites studied here are in their molten state (and thus equilibration of structural and conformational properties at all length scales is easier to achieve). To explore thermodynamic properties, MD simulations were also performed at two other thermodynamic state points: (a) *T* = 393 K and *p* = 1 atm, and (b) *T* = 413 K and *p* = 100 atm. The simulations were carried out using large, cubic simulation cells (edge lengths up to 200 Å) subject to periodic boundary conditions along all three space directions. Technical details regarding the force-field employed for polymer chains and silica nanoparticles, and the simulation runs, can be found in previous studies [21,50,51,52,53]. The simulations were performed with GROMACS (KTH Royal Institute of Technology, Stockholm, Sweden) [54] and lasted for hundreds of nanoseconds.

## 3. Results

### 3.1. Local Density

MD predictions for the density *ρ* of the simulated systems at *T* = 413 K and *p* = 1 atm are shown in Table 2. For pure PEG, the predicted density is 1.015 ± 0.001 g cm^−3^, which agrees well with previously reported experimental [55] and simulation [53] data. At the same thermodynamic conditions, all nanocomposites are characterized by higher densities whose values vary linearly with the concentration of the nanocomposite in silica nanoparticles. At fixed silica volume fraction, on the other hand, the density of the nanocomposites seems to be independent of the size of nanoparticles.

**Figure 2:**
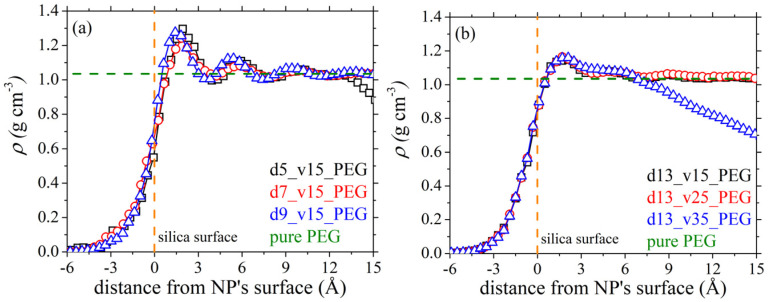
MD predictions (*T* = 413 K and *p* = 1 atm) for the variation of PEG mass density with radial distance from the surface of the silica nanoparticle in the studied nanocomposites, as a function of silica size (**a**), and silica concentration (**b**). The horizontal green dashed line indicates the density of pure PEG at the same thermodynamic conditions, while the perpendicular orange dashed line at *r* = 0 indicates the surface of the nanoparticle.

Figure 2 shows how the polymer mass density around a silica nanoparticle varies as a function of radial distance from the surface of the nanoparticle. For this calculation, we decomposed the space around the nanoparticle in spherical shells, each shell with radius larger than the radius of the previous shell by 0.5 Å; and then we computed the polymer mass inside each one of these shells. The local mass density is then estimated by dividing the polymer mass found in each such shell with the differential volume of that shell. Several sets of results are shown corresponding to different nanoparticle sizes and concentrations in the matrix. In all cases, we recognize the known oscillatory profile already reported in the past for other PEG-silica nanocomposite melts [20,21,24]. Irrespective of the size and loading of the polymer matrix in silica nanoparticles, the local polymer mass density in the vicinity of the nanoparticle increases by almost 20–25% compared to the density of the corresponding bulk PEG melt (1.015 ± 0.001 g cm^−3^). More specifically, the local polymer mass density increases rapidly with increasing distance from the silica surface, reaches a maximum at around 1.8 Å, and then levels off approaching asymptotically the density of pure PEG at the same conditions. For systems d5_v15_PEG and d13_v35_PEG, at even larger distances from the nanoparticle surface, the mass density *ρ* decreases further. This happens because in these systems, the distance between neighboring silica nanoparticles is smaller (Table 3), thus the probability of finding another nanoparticle (instead of bulk polymer mass) next to a reference nanoparticle is higher. An interesting point in all cases discussed in Figure 2 is that the polymer mass density starts to increase at a distance *r* smaller than *r* = 0 (denoting the surface of the nanoparticle), which can be explained by the fact that the silica nanoparticles used in our simulations contain cavities on their surface that are filled by PEG segments, thus allowing for polymer mass to be observed even at distances smaller than the average nanoparticle radius.

### 3.2. Structure and Conformation of Adsorbed and Free PEG Chains

Following previous works [21,46,56], a PEG chain is defined as adsorbed if at least one of its monomers lies inside the adsorbed layer (which here corresponds to the space that extends up to *r* = 3.4 Å from the surface of the nanoparticle). In complete analogy, a PEG chain is considered as non-adsorbed or free if all of its monomers lie outside the adsorbed layer (i.e., at distances beyond 3.4 Å from the surface of the nanoparticle).

MD predictions for the fraction of adsorbed and free chains (obtained from the equilibrated part of the accumulated simulation trajectories) are summarized in Table 3. Clearly, for fixed silica concentration (i.e., 15 *v*/*v*%), the larger the size of the silica nanoparticles, the less the number of PEG chains that adsorb on its surface. This can be explained by calculating the available adsorption area (per chain) on the surface of the nanoparticle (Table 3) at fixed volume fraction in nanoparticles. Then, we see that, with decreasing nanoparticle diameter, the available adsorption surface per polymer chain increases. Interestingly, in the nanocomposite with *d* = 5 nm, this is by ~76% higher than in the nanocomposite with *d* = 9 nm. We draw similar conclusions by examining the dependence of the fraction of adsorbed chains on silica loading.

As explained in detail in our previous study [21], the driving force for the strong adsorption of PEG chains onto silica is the hydrogen bonds (HB) that develop between the polymer and the filler, with the surface silanol groups of silica and the terminal hydroxyl groups of the polymer chains being the donors, and the oxygen atoms of silica and/or PEG chains being the acceptors. How the size and concentration of silica nanoparticles affect the number of these hydrogen bonds in the studied systems is shown in Table 4. For the calculation of the number of hydrogen bonds, well-accepted geometric criteria were applied [57]. In Table 4, we further distinguish between hydrogen bonds that form between the polymer chains of the matrix (denoted as HB_pol-pol_), and hydrogen bonds that form between silica and matrix PEG chains (denoted as HB_pol-sil_). In all cases, the number of hydrogen bonds computed is normalized with the total number of polymer chains in the system.

Clearly, the presence of silica nanoparticles causes a significant increase in the number of hydrogen bonds formed. This is attributed to: (a) the presence of the surface silanol groups on the silica nanoparticles that offer extra donors for the formation of hydrogen bonds with the oxygen atoms of PEG chains, and (b) the surface oxygen atoms of silica that serve as acceptors for the formation of hydrogen bonds with the terminal hydroxyl groups of PEG chains, the latter serving as donors. Interestingly, the number of HB_pol-pol_ in the nanocomposites decreases compared to their value in bulk PEG (= 0.69 hydrogen bonds per chain) due to the preference of the PEG terminal hydroxyl groups to form hydrogen bonds with the silica oxygen atoms (and not with other polymer chains in the matrix). This tendency is more pronounced in the systems with smaller nanoparticles and/or higher silica nanoparticle concentrations. An explanation for this is the higher fraction of adsorbed PEG chains in these systems (Table 3), implying that more PEG chains come into contact with silica nanoparticles. On the other hand, the population of HB_pol-sil_ increases with decreasing nanoparticle diameter and increasing silica content (which is compatible with the larger number of polymer chains coming into contact with silica).

Significant insight into the conformation of the simulated systems can be obtained if one computes the mean-squared end-to-end distance $\langle R_{\mathrm{ee}}^{2}\rangle$ of PEG chains, with the brackets denoting an average over all chains in the simulation cell. From the equilibrated part of the MD trajectories, we have extracted the results that are summarized in Table 5. For the nanocomposites, also the separate $\langle R_{\mathrm{ee}}^{2}\rangle$ values for adsorbed and free polymer chains were computed and reported.

Within the statistical error of the simulations, the overall size of PEG chains remains unaltered by the presence of the nanoparticles. However, a more careful inspection indicates that adsorbed chains are characterized by higher $\langle R_{\mathrm{ee}}^{2}\rangle$ values than PEG chains in their pure melt. This is attributed to the graft-like conformations that adsorbed PEG chains adopt as they develop preferentially away from the nanoparticle surface towards the bulk of the polymer matrix.

**Figure 3:**
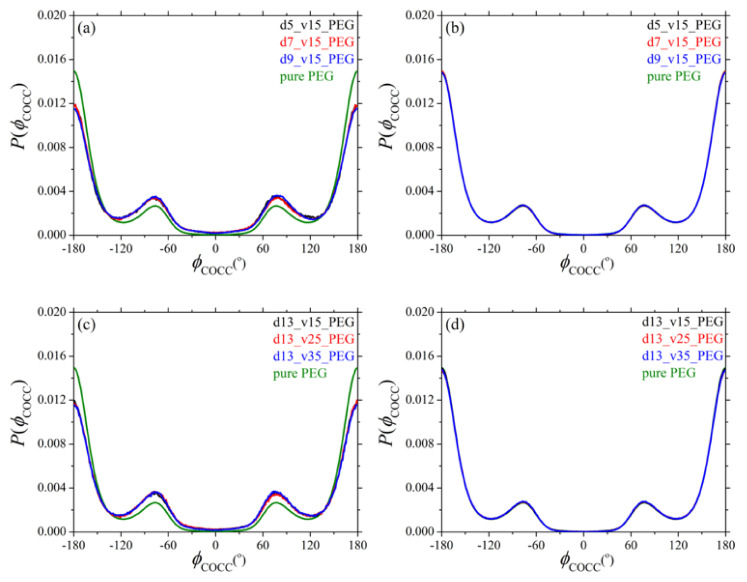
Distribution of the COCC backbone dihedral as obtained from the present *npT* MD simulations at *T* = 413 K and *p* = 1 atm for: (**a**) adsorbed, and (**b**) free chains in systems 2–4 (dependence on nanoparticle size). Parts (**c**,**d**) show the same distributions but for systems 5–7 (dependence on silica loading). In all cases, the green curve corresponds to the result obtained for the pure PEG melt (system 1) at the same thermodynamic conditions.

**Figure 4:**
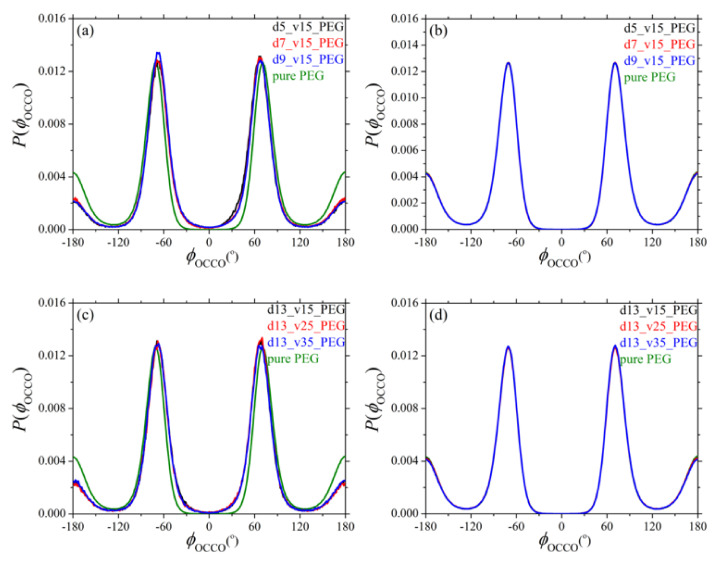
Same as with Figure 3 but for the distribution of the OCCO backbone dihedral. The explanation of (**a**–**d**) is the same as in Figure 3.

Additional information about the conformation of PEG chains in their silica nanocomposites is obtained by analyzing the distributions of the two dihedral angles COCC (*φ*_COCC_) and OCCO (*φ*_OCCO_) along their backbone. The respective MD predictions are presented in Figure 3 and Figure 4, with parts (a) and (b) showing the dependence (for dihedrals in adsorbed and free chain segments, respectively) on silica size, and parts (c) and (d) showing the corresponding dependence on silica loading. Dihedral angles referring to mixed (adsorbed and free) sequences of atoms were not considered in our analysis.

Dihedrals belonging to non-adsorbed (free) segments behave identically as dihedrals in the pure PEG melt. Dihedrals belonging to adsorbed PEG chains, on the other hand, exhibit an enhancement of gauche conformational states (states between −180° and −60°, and between +60° and +180°) indicating that adsorbed PEG chains undergo strong conformational changes as the result of their tight adsorption to the surface of the nanoparticles. A similar finding has been reported by Rissanou et al. [24]. Overall, and irrespective of the size and concentration of silica nanoparticles in the nanocomposites, the distributions are very much the same for the two types of dihedrals (COCC and OCCO) both for adsorbed and non-adsorbed PEG segments.

### 3.3. Static Structure Factor

**Figure 5:**
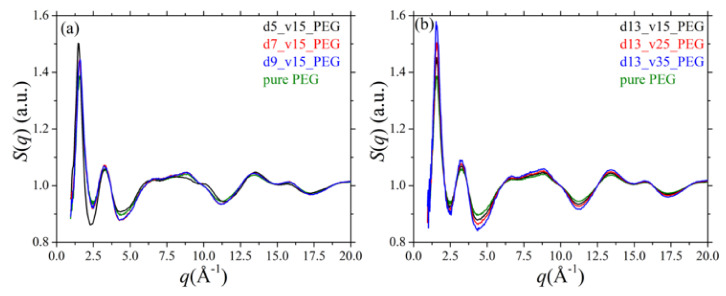
Simulation predictions for the static structure factor *S*(*q*) (*T* = 413 K and *p* = 1 atm) and dependence on: (**a**) silica diameter, and (**b**) silica volume fraction. In all cases, the green curve corresponds to the result obtained for the pure PEG melt (system 1) at the same thermodynamic conditions.

The structure of the PEG chains in all systems studied in the present work can also be examined by calculating the static structure factor, *S*(*q*), which is the Fourier transform of the function *H*(*r*) defined as:

(1) $$H\left(r\right)=\overset{3}{\underset{a=1}{\sum }}\overset{3}{\underset{\beta =1}{\sum }}\frac{x_{a}x_{\beta }f_{a}f_{\beta }}{{\left(\sum_{\gamma =1}^{3}x_{\gamma }f_{\gamma }\right)}^{2}}\left(g_{a\beta }\left(r\right)-1\right)$$

where *x_α_* and *x_β_* denote the number fraction of *α*-type and *β*-type atoms in the system, *f_α_* and *f_β_* the respective scattering factors, and *g_αβ_*(*r*) the total pair distribution function. The values of the scattering factors used in the present work for carbon, hydrogen, and oxygen (the atoms appearing in a PEG chain) can be found in ref [58]. By taking the Fourier transform of the function *H*(*r*),

(2) $$S\left(q\right)=1+\frac{n}{V}\int_{0}^{\infty }4\pi r^{2}\frac{\sin\left(qr\right)}{qr}H\left(r\right)dr$$

where *n*/*V* denotes the total number density of atomistic units, we can compute the diffraction pattern of the pure PEG polymer and of its silica nanocomposites as a function of: (a) silica diameter, and (b) silica concentration; the results are shown in Figure 5a,b, respectively. The first peak in all patterns reflects mainly intermolecular correlations between different polymer chains, whereas the rest of the peaks reflect intrachain correlations. For the pure PEG melt, the positions of the peaks are consistent with previous experimental and MD works [53,59]. Overall, adding silica nanoparticles to the PEG melt causes some non-negligible alterations to the structure of the host chains for all nanocomposites examined. For example, the intensity of the first peak increases in the nanocomposites, implying a more compact arrangement of PEG chains. This becomes more pronounced as the size of the nanoparticles decreases or as their concentration in the nanocomposite increases, because the fraction of adsorbed PEG chains that adsorb onto silica nanoparticles increases. On the other hand, the peaks at larger *q* values (reflecting intramolecular correlations) become more diffuse, which is related to the conformational changes of adsorbed PEG chains (as also recorded in the distribution of the two backbone dihedral angles shown in Figure 3 and Figure 4). Again, these conformational changes become more pronounced as the size of the silica nanoparticles decreases or as their volume fraction in the nanocomposite increases.

### 3.4. Single Chain form Factor

Another quantity that provides important information about the internal structure of a polymer chain is the single chain form factor, *P*(*q*), defined according to the following equation:

(3) $$P\left(q\right)=\frac{1}{\sum_{k=1}^{N_{ch}}\sum_{i=1}^{N}\sum_{j=1}^{N}f_{i}f_{j}}\overset{N_{ch}}{\underset{k=1}{\sum }}\overset{N}{\underset{i=1}{\sum }}\overset{N}{\underset{j=1}{\sum }}\langle f_{i}f_{j}\frac{\sin\left(qr_{ij}\right)}{qr_{ij}}\rangle$$

where *N_ch_* and *N* denote the number of PEG chains in the system and the number of atoms in each chain, *f_i_* and *f_j_* the respective scattering factors, and *r_ij_* the distance between atoms *i* and *j* along the same chain. The brackets on the right-hand side indicate a configurational average over all PEG chains in the nanocomposite (adsorbed and free). For the pure PEG melt, the MD results for the single chain form factor can be compared with the analytical expression derived by Burchard and Kajiwara [60] for the random-flight model, which assumes randomly oriented segments along the chain:

(4) $$P_{r-f}\left(q\right)=\frac{2}{N_{K}^{2}}\left[\frac{N_{K}}{1-\frac{\sin\left(qb\right)}{qb}}-\frac{N_{K}}{2}-\frac{1-{\left(\frac{\sin\left(qb\right)}{qb}\right)}^{N_{K}}}{{\left(1-\frac{\sin\left(qb\right)}{qb}\right)}^{2}}\frac{\sin\left(qb\right)}{qb}\right]$$

**Figure 6:**
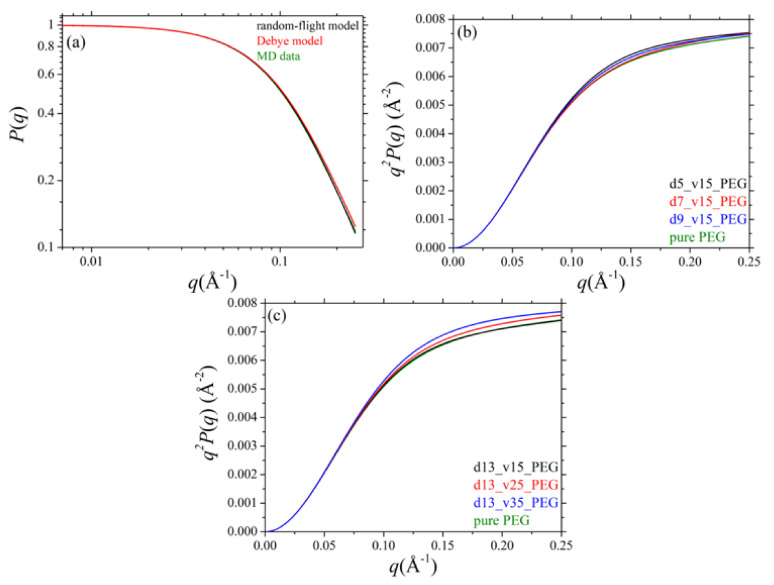
(**a**) MD predictions for the single chain form factor, *P*(*q*), of pure PEG melt (*T* = 413 K and *p* = 1 atm), and the respective predictions provided by the random-flight model and the Debye equation. (**b**) Kratky plots (*q^2^P*(*q*) versus *q*) for all systems studied (pure PEG and PEG-silica nanocomposites) and dependence on silica nanoparticle size. (**c**) Kratky plots (*q^2^P*(*q*) versus *q*) for all systems studied (pure PEG and PEG-silica nanocomposites) and dependence on the volume fraction of nanoparticles.

Here, *N_K_* denotes the number of Kuhn segments per chain and *b* the Kuhn length (equal to 5.95 Å for the PEG matrix chains studied here). For very long Gaussian chains, the single chain form factor is also described by the Debye function [61]:

(5) $$P_{D}\left(q\right)=\frac{2}{{\left(R_{g}q\right)}^{4}}\left[e^{-{\left(R_{g}q\right)}^{2}}-1+{\left(R_{g}q\right)}^{2}\right]$$

where *R*_g_ denotes the mean radius of gyration for the chains. The single chain form factor calculated by the MD simulations (Figure 6a) is in excellent agreement with the predictions of the random-flight model. Although the Debye equation is valid for quite long chains, its predictions are also in good agreement with the MD results except from some slight deviations at higher *q* values.

The corresponding results for the nanocomposites are analyzed in parts b and c of Figure 6 showing the so-called Kratky plots, i.e., the *q^2^P*(*q*) curves versus *q*. Figure 6b shows the dependence on silica diameter and Figure 6c the dependence on silica volume fraction. No deviations are observed at low *q* values. At higher *q* values, on the other hand, deviations are observed which are correlated with the fraction of adsorbed PEG chains on the silica nanoparticles and the resulting alterations in the structure of adsorbed polymer chains. The deviations become more pronounced as the size of the silica nanoparticles decreases (at fixed volume fraction) or as their volume fraction in the nanocomposite increases (i.e., as the relative fraction of adsorbed PEG chains increases).

### 3.5. Network of Nanoparticle Bridging Chains

Of particular interest for future studies of the rheological and mechanical properties of the simulated attractive PEG-silica nanocomposites is the quantification of the structure of the underlying network that forms as PEG chains adsorb simultaneously at two different nanoparticles, effectively playing the role of bridges. As already mentioned in the introduction, a key parameter that dictates the formation of this network is the average inter-particle (face-to-face) separation *d*_inter_. The values of *d*_inter_ for each of the six nanocomposite melts studied in the present work have been listed in Table 3. Clearly, with decreasing nanoparticle size and increasing volume fraction, *d*_inter_ decreases, thus favoring the formation of a network between nanoparticles.

**Figure 7:**
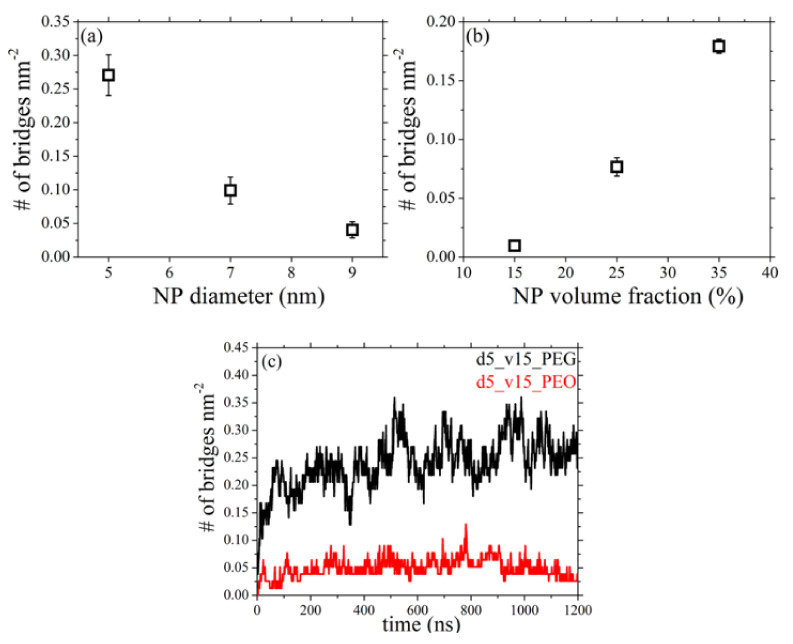
Number of polymer bridges per silica nm^2^ from the present *npT* MD simulations (*T* = 413 K and *p* = 1 atm) as a function of: (**a**) nanoparticle diameter (systems 2–4), and (**b**) nanoparticle volume fraction (systems 5–7). (**c**) Time evolution of the number of bridges formed per silica nm^2^ in the d5_v15_PEG and d5_v15_PEO nanocomposites.

Figure 7 depicts the number of bridges (*n*_b_) formed as a function of silica diameter and silica volume fraction (the results are shown normalized with the available surface nanoparticle area for adsorption in each melt, see third column in Table 3). Because of the use of only one nanoparticle in our simulations, to compute the number of polymer bridges we identified those PEG chains that adsorb simultaneously to the nanoparticle inside the primary cell and to its images in the 26 neighboring cells. According to Figure 7a, a decrease in the size of the silica nanoparticle causes a significant increase in the number of bridges formed in the melt; this implies that, at fixed silica loading, smaller particles enhance network formation, which agrees perfectly with the experimental studies for P2VP/silica nanocomposites [16]. The same holds if one increases the volume fraction (Figure 7b) because of the smaller *d*_inter_ values characterizing nanocomposites containing higher silica concentrations, thereby making it easier for PEG chains to extend out from a given nanoparticle and to adsorb to another one.

**Figure 8:**
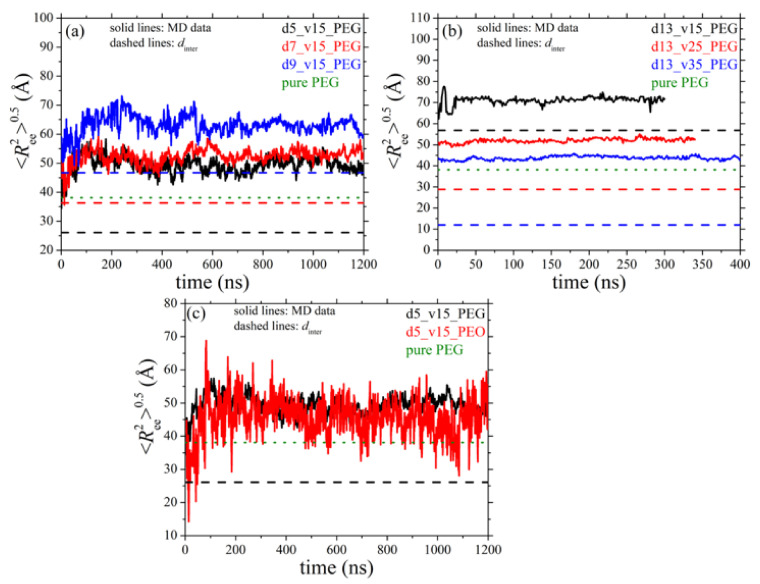
Time evolution of the end-to-end distance $\langle R_{\mathrm{ee}}^{2}\rangle {}^{0.5}$ of bridging chains as a function of: (**a**) nanoparticle size (systems 2–4), (**b**) nanoparticle volume fraction (systems 5–7), and (**c**) type of PEG chain terminal units (systems 2 and 8), as obtained from the present *npT* MD simulations (*T* = 413 K and *p* = 1 atm). The solid line in each graph represents the simulation data, the horizontal dashed line indicates the *d*_inter_ value for the corresponding nanocomposite, and the green horizontal dotted line indicates the $\langle R_{\mathrm{ee}}^{2}\rangle {}^{0.5}$ value of PEG chains in their pure melt at the same thermodynamic conditions (Table 5).

**Figure 9:**
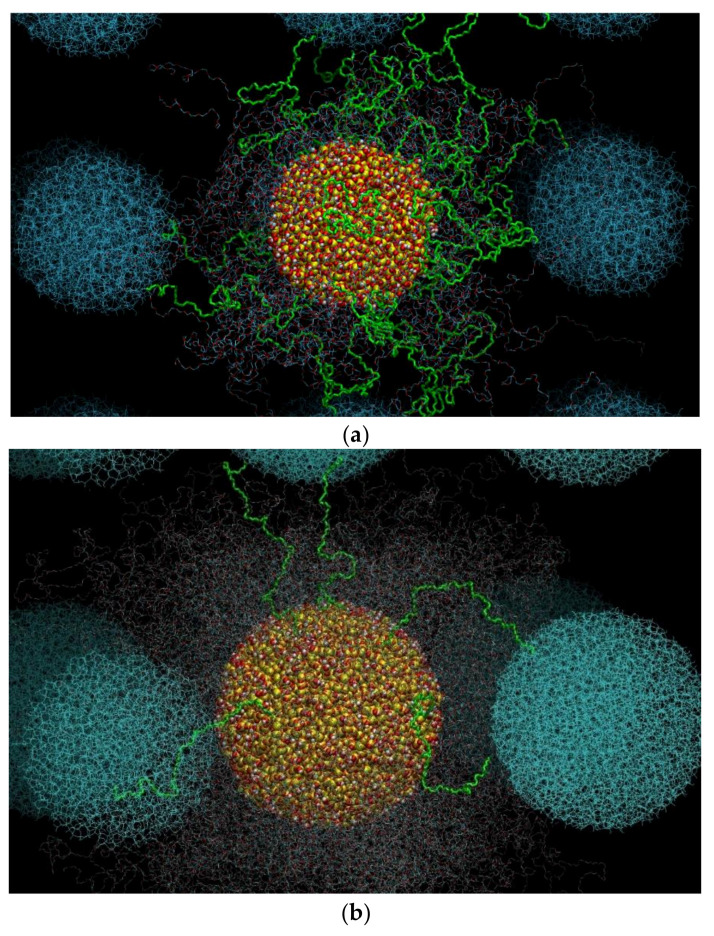
Typical atomistic snapshots of the nanoparticle network formed in: (**a**) the d5_v15_PEG, and (**b**) the d9_v15_PEG nanocomposite (*T* = 413 K and *p* = 1 atm). With yellow, red, and white we show the silica atoms. With blue we indicate images of the silica nanoparticle in neighboring cells. Non-bridging chains are shown in red, white, and cyan, and bridging ones in green.

The necessary extension of PEG chains to develop bridges between different nanoparticles can be quantified by examining the size of bridging chains. Figure 8 shows the time evolution of $\langle R_{\mathrm{ee}}^{2}\rangle {}^{0.5}$ for bridging chains as a function of silica diameter and silica loading. In the figure we also indicate with the dashed lines the value of *d*_inter_ for each nanocomposite system (Table 3), whereas the dotted lines denote the $\langle R_{\mathrm{ee}}^{2}\rangle {}^{0.5}$ values of PEG chains in their own melt (Table 5). Surprisingly, apart from those nanocomposites where *d*_inter_ is smaller than the $\langle R_{\mathrm{ee}}^{2}\rangle {}^{0.5}$ of the pure PEG (systems 2, 3, 6 and 7), and thus a network is expected to develop, PEG chains appear to form bridges even in the nanocomposites where $\langle R_{\mathrm{ee}}^{2}\rangle {}^{0.5}$ is considerably larger than its bulk value (e.g., systems 4 and 5). In all cases, bridging PEG chains assume highly extended conformations as they are simultaneously adsorbed on different nanoparticles, a behavior which is more pronounced in the systems containing larger nanoparticles or characterized by lower nanoparticle concentrations (i.e., when *d*_inter_ increases). Interestingly, for the system with the larger silica nanoparticle and the lowest volume fraction (d13_v15_PEG), the value of $\langle R_{\mathrm{ee}}^{2}\rangle {}^{0.5}$ of bridging chains is almost two times larger compared to the unperturbed polymer size. Typical atomistic snapshots of the network of bridging chains formed in systems 2 and 4 are depicted in Figure 9, revealing the denser structure of the network in the nanocomposite with the smaller-sized particles (i.e., *d* = 5 nm).

The effect on the formation of the network when the hydroxyl terminal groups of the host PEG chains are replaced by methoxy ones has also been examined. To this, we compare the number of bridges per unit nanoparticle surface area and the value of $\langle R_{\mathrm{ee}}^{2}\rangle {}^{0.5}$ of the bridging chains between systems d5_v15_PEG and d5_v15_PEO, and the MD predictions are presented in part (c) of Figure 7 and Figure 8, respectively. It appears that the chemistry of the terminal units of the host polymer chains plays a key role for the development of the bridging network in the studied PEG-silica nanocomposites. More specifically, *n*_b_ becomes almost five times smaller when methoxy groups serve as terminal units instead of hydroxyl groups (Figure 7c). This is clearly related with the different adsorption mechanisms of the two different polymers, as thoroughly discussed in a recent study [21]. Hydroxyl-terminated PEG chains tend to adsorb laterally on the silica surface thus forming graft-like conformations, which makes it easier for them to extend to longer distances and form a bridge with another silica nanoparticle. Methoxy-terminated chains, on the other hand, adsorb tightly onto the silica nanoparticle along their entire contour (they practically lie on the nanoparticle surface), a behavior which prohibits contacts with other nanoparticles. However, upon examining the size of bridging chains, it appears that, within statistical error, the value of $\langle R_{\mathrm{ee}}^{2}\rangle {}^{0.5}$ of PEG chains in the two nanocomposites is the same, implying that at fixed nanoparticle size and volume fraction and irrespective of the end units of the matrix chains, these should extend by the same factor in order to adsorb to another silica nanoparticle. That this nanoparticle network formation is less favored in the case of the d5_v15_PEO nanocomposite is further implied by the strong fluctuations seen in the $\langle R_{\mathrm{ee}}^{2}\rangle {}^{0.5}$-vs.-*t* curve in Figure 8c for this system, which is due to the smaller number of PEO chains that bridge silica nanoparticles compared to PEG ones under exactly the same conditions of loading of the nanocomposite in nanoparticles (see also Figure 7c). To the best of our knowledge, the dependence of network formation on the nature of polymer terminal units has not been discussed in the literature before. The corresponding dependence on the MW of the polymer matrix chains will be the subject of a future contribution.

### 3.6. Orientational Relaxation of Polymer Chains

**Figure 10:**
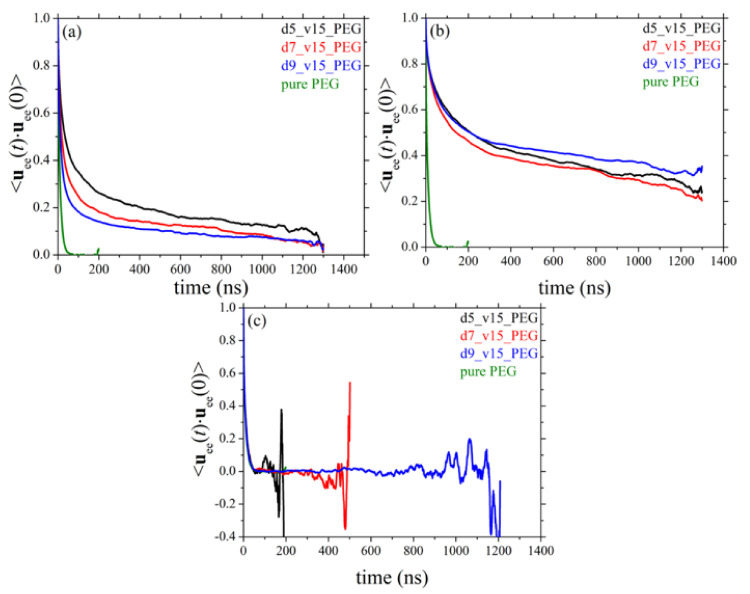
Decay of the ACF of the chain end-to-end unit vector in time (*T* = 413 K and *p* = 1 atm) in systems 1–4 (dependence on silica size). The results refer to: (**a**) all chains, (**b**) only adsorbed chains, and (**c**) only free chains in the corresponding nanocomposites.

**Figure 11:**
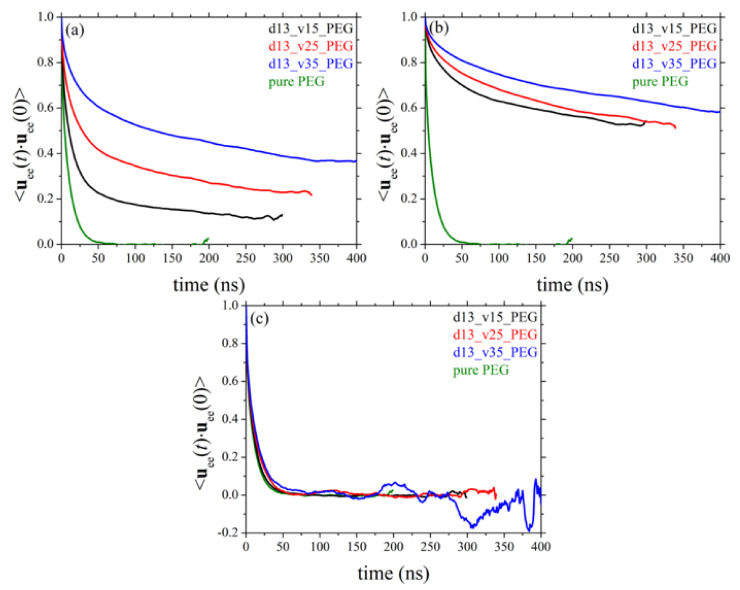
Same as with Figure 10 but for systems 1, 5, 6, and 7 (dependence on silica concentration). The explanation of (**a**–**c**) is the same as in Figure 10.

Chain terminal relaxation can be quantified by calculating the decay of the time autocorrelation function (ACF) of the chain end-to-end unit vector denoted as ⟨**u**_ee_(*t*)·**u**_ee_(0)⟩. The decay of ⟨**u**_ee_(*t*)·**u**_ee_(0)⟩ for the pure PEG melt and its silica-based nanocomposites as a function of nanoparticle size and nanoparticle loading is presented in Figure 10 and Figure 11, respectively. Part (a) of the Figures depicts the ACF curves for the entire population of PEG chains in the various nanocomposites; parts (b) and (c), on the other hand, present the respective ACF functions separately for adsorbed and free chains in the given system. In all cases, the corresponding relaxation curve of PEG chains in their own melt is shown with the green curve. From Figure 10a it becomes clear that the presence of the silica nanoparticles considerably slows down the orientational dynamics of polymer chains due to their strong attractive interactions with the nanoparticles. Decreasing the size of nanoparticles or increasing their concentration in the melt further slows down dynamics, which is related to the higher fraction of adsorbed PEG chains in the nanocomposites with smaller-sized nanoparticles or higher nanoparticle volume fractions (Table 3). As expected, orientational deceleration is more pronounced in the case of adsorbed PEG chains (Figure 10b); for these chains, the corresponding ACF curves deviate significantly from the corresponding bulk behavior as they tend to approach a plateau at long times implying a too slow relaxation to be tracked ergodically by the MD simulation. As far as the orientational relaxation of non-adsorbed chains is concerned (Figure 10c), although full relaxation in all cases is observed, the corresponding curves are above that corresponding to the pure polymer. This is more pronounced in the nanocomposites where nanoparticles have a smaller diameter or are present in the melt in higher concentrations, and is attributed to the polymer network formed that severely constrains the orientational motion of non-adsorbed PEG chains.

### 3.7. Diffusive Behavior of Polymer Chains

**Figure 12:**
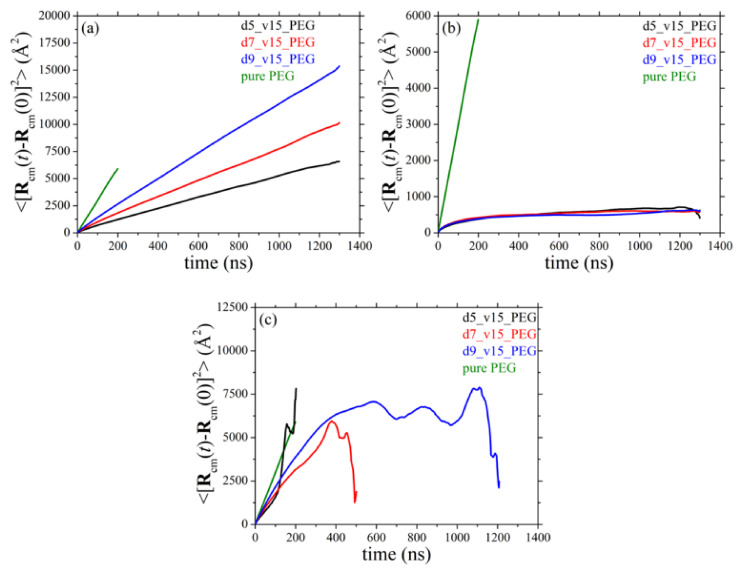
Mean-square displacement of the chains centers-of-mass as a function of time for: (**a**) all PEG chains, (**b**) only adsorbed PEG chains, and (**c**) only free PEG chains (*T* = 413 K and *p* = 1 atm) in systems 1–4 (dependence on silica size).

**Figure 13:**
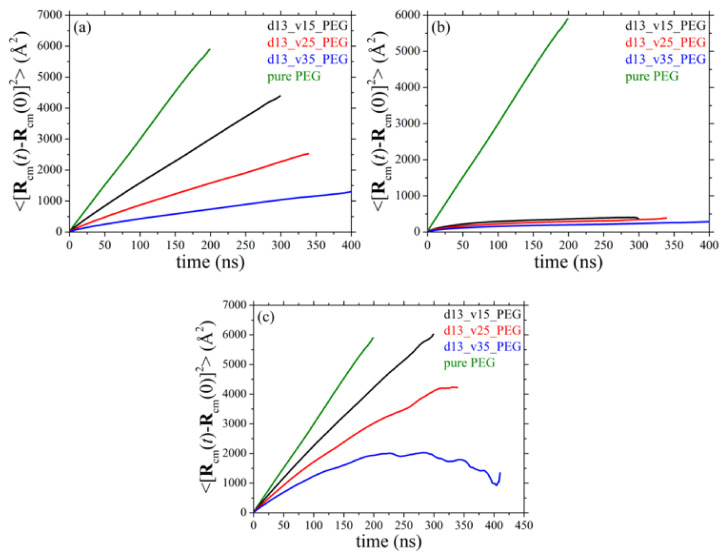
Same as with Figure 12 but for systems 1, 5, 6, and 7 (dependence on silica concentration). The explanation of (**a**–**c**) is the same as in Figure 12.

We discuss next the simulation results for the mean-square displacement (msd) of the chains centers-of-mass $\langle {\left[R_{\mathrm{cm}}\left(t\right)-R_{\mathrm{cm}}\left(0\right)\right]}^{2}\rangle$ with time *t* in the simulated systems. More specifically, Figure 12 and Figure 13 present the msd curves for all systems examined as a function of silica diameter and silica concentration, respectively, averaged over all PEG chains (Figure 12a and Figure 13a), over only adsorbed PEG ones (Figure 12b and Figure 13b), and over only free PEG chains (Figure 12c and Figure 13c). As before, for direct comparison, in each figure we have included the corresponding result for PEG chains in their own melt (green curve). The presence of silica nanoparticles significantly decelerates the overall translational motion of the full population of matrix chains. We also see that adsorbed PEG chains (Figure 12b and Figure 13b) exhibit only features of local dynamics in the vicinity of the nanoparticle with their long-time diffusive motion being highly suppressed. Similar to the results for the orientational dynamics of the host matrix chains, the MD data suggest that the translational motion of non-adsorbed chains in all nanocomposites (Figure 12c and Figure 13c) is dramatically restricted compared to the pure PEG melt. The degree of deceleration is again larger in the nanocomposites containing either smaller-sized nanoparticles or nanoparticles in higher concentrations; these are the systems where the polymer network that forms between nanoparticles and PEG chains is denser, rendering the polymer matrix more rigid, thus putting significant constraints to the spatial displacement also of free chains.

### 3.8. Dynamic Structure Factor

**Figure 14:**
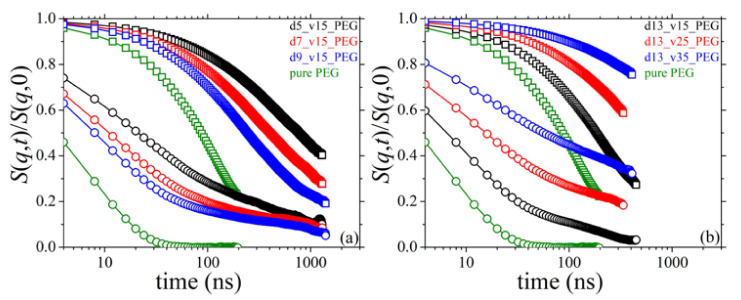
MD-predicted *S*(*q*,*t*)/*S*(*q*,0)-vs.-*t* spectra (*T* = 413 K and *p* = 1 atm) and dependence on: (**a**) silica size, and (**b**) silica concentration. Results are shown for *q* = 0.04 Å^−1^ (open squares) and *q* = 0.15 Å^−1^ (open circles).

We turn our attention next to the calculation of the single chain intermediate coherent dynamic structure factor *S*(*q*,*t*), which practically reflects the Brownian motion of polymer chains in their melt, and which can be experimentally accessed through state-of-the-art techniques such as neutron spin echo (NSE) and dynamic light scattering (DLS). Given an MD trajectory for an isotropic system, *S*(*q*,*t*) is computed through:

(6) $$S\left(q,t\right)=\frac{1}{\sum_{i=1}^{N_{\mathrm{ch}}}\sum_{n=1}^{N}f_{i,n}^{2}}\overset{N_{\mathrm{ch}}}{\underset{i=1}{\sum }}\overset{N}{\underset{n=1}{\sum }}\overset{N}{\underset{m=1}{\sum }}\langle f_{i,n}f_{i,m}\frac{\sin\left(qR_{i,nm}\left(t\right)\right)}{qR_{i,nm}\left(t\right)}\rangle$$

where *N*_ch_ denotes the total number of chains in the system, *q* is the magnitude of the scattering vector **q**, *f_i_*_,*n*_ and *f_i_*_,*m*_ are the scattering factors of atoms *n* and *m* along the same chain *i*, *R_i_*_,*nm*_(*t*) is the magnitude of the displacement vector **R***_i_*_,*nm*_(*t*) = **R***_i_*_,*n*_(*t*) − **R***_i_*_,*m*_(*t*) between atoms *n* and *m* on chain *i*, and the brackets denote a configurational average. MD simulation results for the ratio *S*(*q*,*t*)/*S*(*q*,0) for the bulk PEG melt and its silica-based nanocomposite melts studied here are shown in Figure 14 revealing significant differences between the various systems, with the *S*(*q*,*t*)/*S*(*q*,0) spectra for the nanocomposites being well above those for the pure melt and decaying much slower. This is obvious not only for the small *q* value examined (*q* = 0.04 Å^−1^, open squares in Figure 14) reflecting the diffusive behavior of PEG chains, but also for the large one (*q* = 0.15 Å^−1^, open circles in Figure 14) reflecting local dynamics. The slow-down in the dynamics (practically at all length scales examined) is more pronounced in the nanocomposites with the smaller silica nanoparticles (Figure 14a) or the higher silica concentration (Figure 14b) (all other properties kept the same), which is fully consistent with the conclusions drawn from the analysis of orientational and diffusive dynamics already discussed in previous sections.

### 3.9. Self-Intermediate Scattering Function

**Figure 15:**
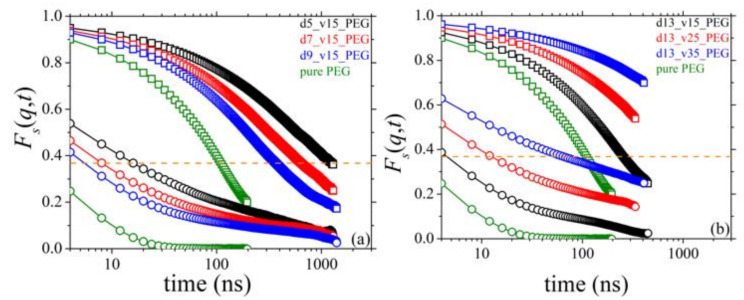
MD-predicted *F_s_*(*q*,*t*)-vs.-*t* spectra (*T* = 413 K and *p* = 1 atm) and dependence on: (**a**) silica size, and (**b**) silica concentration. Results are shown for *q* = 0.04 Å^−1^ (open squares) and *q* = 0.15 Å^−1^ (open circles). The orange dashed line indicates the value 1/*e* of the self-intermediate scattering function (attained when time equals the characteristic relaxation time).

We have also computed the self-intermediate scattering function *F_s_*(*q*,*t*), which focuses on time-dependent spatial variations of the dynamics of single atoms. The self-intermediate scattering function, which is experimentally measured by adopting incoherent neutron scattering techniques, reflects the impact of interactions of single PEG atoms with the silica nanoparticles on their dynamics. From MD, *F_s_*(*q*,*t*) is calculated through the equation:

(7) $$F_{s}\left(q,t\right)=\frac{1}{\sum_{i=1}^{N_{\mathrm{ch}}}\sum_{n=1}^{N}f_{i,n}}\overset{N_{\mathrm{ch}}}{\underset{i=1}{\sum }}\overset{N}{\underset{n=1}{\sum }}\langle f_{i,n}\frac{\sin\left(qR_{i,n}\left(t\right)\right)}{qR_{i,n}\left(t\right)}\rangle$$

where *R_i_*_,*n*_(*t*) is the magnitude of the displacement vector **R***_i_*_,*n*_(*t*) = **R***_i_*_,*n*_(*t*) − **R***_i_*_,n_(0) of atom *n* on chain *i*. The MD simulation results for *F_s_*(*q*,*t*) for the simulated nanocomposites are shown in Figure 15, including a direct comparison with those for the pure PEG melt. Qualitatively, the results are very similar to those for *S*(*q*,*t*)/*S*(*q*,0), revealing again strong deviations in the dynamics between nanocomposites and pure PEG melt. More specifically, the *F_s_*(*q*,*t*) curves for all nanocomposites are above the corresponding ones for the bulk PEG melt and decay much slower. This deceleration in the dynamics is true for both values of *q* studied: *q* = 0.04 Å^−1^ (open squares in Figure 15) and *q* = 0.15 Å^−1^ (open circles in Figure 15), reflecting the impact of PEG chain-silica nanoparticle interactions on the single atom dynamics at long and short scales, respectively. Dynamics is more suppressed in the nanocomposites containing smaller nanoparticles (Figure 15a) at fixed loading or nanoparticles (of given diameter) at higher concentrations (Figure 15b). These behaviors are consistent with all previous conclusions and can be explained by the respective alterations in the fraction of absorbed polymer chains and the number of PEG chains bridging different nanoparticles. The characteristic long relaxation time is defined as the time *τ* for which *F_s_*(*q*,*τ*)=1/*e* [13], and is denoted with the horizontal dashed orange line in Figure 15a,b. For *q* = 0.15 Å^−1^, in particular, Figure 15b indicates that dynamics slows down at long times; the *F_s_*(*q*,*t*) curves do not seem to drop to zero, which must be associated with the nanoparticle network that forms in the melt, mediated by bridging PEG chains.

### 3.10. Thermal Expansion Coefficient and Isothermal Compressibility

**Figure 16:**
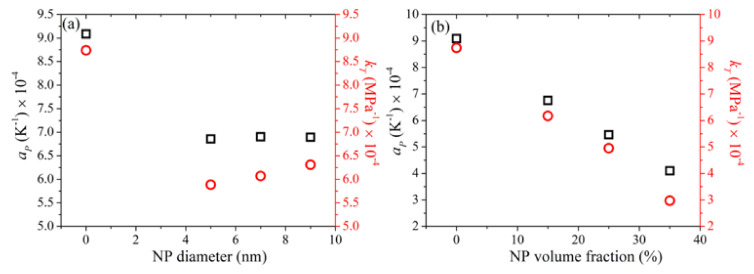
MD predictions for the thermal expansion coefficient *a_P_* (black color) and isothermal compressibility *k_T_* (red color) of the simulated nanocomposites, and dependence on nanoparticle size (**a**), and nanoparticle volume fraction (**b**).

In the last part of the work, we examined how the presence of silica nanoparticles affects the thermodynamic properties of the host PEG matrix, and how this effect depends on silica nanoparticle diameter and degree of nanoparticle loading in the matrix. To this, we have calculated the thermal expansion coefficient (*a_p_*) defined as:

(8) $$a_{p}=\frac{1}{\langle V\rangle }{\left.\frac{\Delta \langle V\rangle }{\Delta T}\right|}_{p}$$

and the isothermal compressibility (*k_T_*) defined as:

(9) $$k_{T}=-\frac{1}{\langle V\rangle }{\left.\frac{\Delta \langle V\rangle }{\Delta p}\right|}_{T}$$

for all simulated systems, where the symbols *V*, *T*, and *p* stand for volume, temperature, and pressure, respectively. For these calculations, two extra MD simulations were performed, one at *T* = 393 K and *p* = 1 atm for the calculation of the thermal expansion coefficient, and a second one at *T* = 413 K and *p* = 100 atm for the calculation of the isothermal compressibility. The corresponding predictions are reported in Figure 16 with the black-colored symbols referring to *a_p_* and the red-colored ones to *k_T_*. Part (a) of the figure shows the dependence on nanoparticle diameter and part (b) the dependence on nanoparticle loading.

For the pure PEG melt, the values of *a_P_* and *k_T_* predicted from the simulations are 9.07 × 10^−4^ K^−1^ and 8.73 × 10^−4^ MPa^−1^, respectively, which agree quite well with experimentally measured ones under similar conditions: *a_P_* = 7.6 × 10^−4^ K^−1^ [62] and *k_T_* = 7.7 × 10^−4^ MPa^−1^ [55]. For the PEG-silica nanocomposites, our simulations reveal a significant decrease in both *a_P_* and *k_T_*, under all circumstances. Both thermodynamic quantities show a strong dependence on silica concentration with their values decreasing almost linearly with increasing nanoparticle volume fraction (Figure 16b). On the other hand, the dependence on the size of the silica nanoparticles (Figure 16a) is milder: *a_P_* is not affected much, whereas *k_T_* exhibits a mild decrease with decreasing nanoparticle diameter. As shown in Table 3, a decrease in the size of the silica nanoparticles causes an increase in the amount of PEG adsorbed on silica. These adsorbed regions are characterized by higher densities than the bulk ones (Figure 2) and are less compressible, and this explains why *k_T_* decreases slightly with decreasing nanoparticle diameter.

## 4. Conclusions

The MD results presented in this work provide detailed information concerning the structure, conformation, underlying topological network between nanoparticles and tie polymer chains, dynamic, and thermodynamic properties of PEG-silica nanocomposites, with an emphasis on the role of the adsorbed (interfacial) polymer layer and the chemistry of end-functional groups.

The local polymer mass density radially from the surface of the silica nanoparticles is higher (up to ~25%) than the density of the pure PEG melt at the same thermodynamic conditions, typical for polymers adsorbed onto solid surfaces. In contrast to PE-silica nanocomposites [33], the local density around the silica nanoparticles does not decrease with increasing curvature (decreasing silica diameter), at least for the range of diameters examined here. The fraction of polymer chains adsorbed onto silica increases significantly with decreasing nanoparticle radius and increasing nanoparticle volume fraction due to the higher surface area available for adsorption in the systems with the smaller particles and the higher loadings.

For all PEG-silica nanocomposites examined, the MD simulations reveal an increase in the size (average mean-squared end-to-end distance) of adsorbed polymer chains compared to their own melt, which is attributed to the graft-like conformation that these chains adopt upon adsorption on the silica nanoparticle [21]. This graft-like nature of the adsorbed PEG chains is also the driving force for the development of a nanoparticle network in the nanocomposites, with silica nanoparticles acting as anchoring points and polymer chains as bridges connecting neighboring nanoparticles. Apart from elucidating the effects of nanoparticle size and loading of the nanocomposite in nanoparticles, our work reveals the key role played by the specific chemistry of the end units of PEG chains in such nanocomposites. By switching from hydroxyl-terminated to methoxy-terminated chains, the formation of the network is suppressed, since the relative population of bridging chains decreases (a direct result of their different mechanisms of adsorption on the silica nanoparticles), which is reported here for the first time.

By analyzing specific time autocorrelation functions, we found that both the orientational and the translational dynamics of PEG chains in their nanocomposites are significantly constrained, as it has also been reported in the past for attractive [20,31] and non-attractive [25,39,40,44] silica-based polymer nanocomposites. The orientational dynamics of non-adsorbed chains are also affected (they slow down), particularly in the nanocomposites containing smaller-sized nanoparticles or higher silica concentrations, due to the denser nanoparticle network that develops in these systems. Similar conclusions were drawn by monitoring the mean-square displacement (msd) of PEG chains centers-of-mass. We also computed the dynamic structure factor *S*(*q*,*t*) and the self-intermediate scattering function *F_s_*(*q*,*t*), quantities that can be accessed through state-of-the-art experimental techniques, for all simulated melts (pure PEG and PEG-silica nanocomposites).

By extending our simulations to lower temperatures and higher pressures, we obtained estimates of the thermal expansion coefficient and isothermal compressibility. For the bulk PEG melt, good agreement with experimental literature data was found. In the nanocomposites, the values of both of these important thermodynamic quantities are considerably lower, dropping almost linearly with the concentration of the nanocomposite in nanoparticles. In contrast, the two thermodynamic quantities are less sensitive to the size of the nanoparticles: with increasing nanoparticle size (keeping the nanoparticle concentration constant), we find that the value of *a_P_* remains practically constant while that of *k_T_* exhibits a small increase.

Future work should address the dependence of microscopic dynamics on the size, concentration, and surface concentration of the nanoparticles in silanol groups, as well as the effect of the molecular weight of the polymer matrix on the statistical properties of the underlying topological network formed (potentially, longer chains should be able to link more nanoparticles). It will also be of interest to investigate the rheological properties of these systems in order to quantify the effect of such a nanoparticle network on the viscosity of the nanocomposite melt, but also to examine the conditions under which the shear flow can drive chain desorption from the nanoparticles.

## Figures and Tables

**Table 1:** Molecular characteristics of the simulated systems.

System	Abbreviation	Number of PEG Chains	Silanol Concentration (OH nm^−2^)	Volume Fraction (*v*/*v*%)	Silica Nanoparticle Diameter (nm)	Total Number of Interacting Atoms
1	PEG	1000	-	-	-	126,000
2	d5_v15_PEG	130	3.6	15	5	21,160
3	d7_v15_PEG	354	3.6	15	7	57,636
4	d9_v15_PEG	750	3.6	15	9	122,201
5	d13_v15_PEG	1799	4.2	15	12.8	305,887
6	d13_v25_PEG	975	4.2	25	12.8	202,063
7	d13_v35_PEG	600	4.2	35	12.8	154,813
8	d5_v15_PEO	130	3.6	15	5	21,940

**Table 2:** Density of all PEG-silica systems studied in the present work (*T* = 413 K and *p* = 1 atm).

System	*ρ* (g cm^−3^)
PEG	1.015 ± 0.001
d5_v15_PEG	1.247 ± 0.004
d7_v15_PEG	1.241 ± 0.002
d9_v15_PEG	1.240 ± 0.002
d13_v15_PEG	1.264 ± 0.001
d13_v25_PEG	1.437 ± 0.001
d13_v35_PEG	1.602 ± 0.001

**Table 3:** MD predictions for the fraction of adsorbed and free chains in the simulated nanocomposites (systems 2–7) at *T* = 413 K and *p* = 1 atm. In the third column of the table, we report the available adsorption surface area per PEG chain. The fourth column denotes the inter-particle distance *d*_inter_ in each nanocomposite examined. ^a^ Data obtained from ref. [21].

System	Fraction of		Available Adsorption Surface per PEG Chain (nm^2^ chain^−1^)	Interparticle Distance *d*_inter_ (nm)
	Adsorbed PEG Chains	Free PEG Chains		
d5_v15_PEG	0.779 ± 0.021	0.221 ± 0.021	0.597	2.61
d7_v15_PEG	0.601 ± 0.013	0.399 ± 0.013	0.434	3.63
d9_v15_PEG	0.424 ± 0.008	0.576 ± 0.008	0.339	4.67
d13_v15_PEG	0.346 ± 0.006 ^a^	0.654 ± 0.006 ^a^	0.285	5.68
d13_v25_PEG	0.598 ± 0.005	0.402 ± 0.005	0.526	2.88
d13_v35_PEG	0.781 ± 0.006	0.219 ± 0.006	0.855	1.20

**Table 4:** MD simulation predictions for the number of hydrogen bonds formed in the simulated PEG-silica nanocomposites per PEG chain present in the melt (*T* = 413 K and *p* = 1 atm). For the nanocomposites, separate results are reported for HB_pol-pol_ and HB_pol-sil_.

System	Hydrogen Bonds Per Chain		
	Total	Polymer-Polymer (HB_pol-pol_)	Polymer-Silica (HB_pol-sil_)
PEG	0.690	0.690	-
d5_v15_PEG	1.416	0.559	0.857
d7_v15_PEG	1.258	0.568	0.690
d9_v15_PEG	1.073	0.621	0.451
d13_v15_PEG	1.072	0.614	0.458
d13_v25_PEG	1.394	0.557	0.838
d13_v35_PEG	1.742	0.497	1.245

**Table 5:** MD predictions for the $\langle R_{\mathrm{ee}}^{2}\rangle$ of PEG chains (*T* = 413 K and *p* = 1 atm). As also explained in the text, for the nanocomposites, we also report the separate $\langle R_{\mathrm{ee}}^{2}\rangle$ values for adsorbed and free PEG chains. ^a^ Data obtained from ref. [21].

System	$\langle R_{\mathrm{ee}}^{2}\rangle$(Å^2^)		
	Adsorbed PEG Chains	Free PEG Chains	All PEG Chains
PEG	-	-	1454 ± 31 ^a^
d5_v15_PEG	1518 ± 36	1379 ± 68	1479 ± 30
d7_v15_PEG	1580 ± 29	1380 ± 41	1501 ± 25
d9_v15_PEG	1556 ± 55	1415 ± 47	1475 ± 38
d13_v15_PEG	1587 ± 42 ^a^	1443 ± 23 ^a^	1487 ± 18 ^a^
d13_v25_PEG	1544 ± 40	1395 ± 55	1482 ± 30
d13_v35_PEG	1495 ± 38	1346 ± 93	1432 ± 34

## Data Availability

The data presented in this study are available upon request from the corresponding author.
